# Resolving the Contradictory Functions of Lysine Decarboxylase and Butyrate in Periodontal and Intestinal Diseases

**DOI:** 10.3390/jcm10112360

**Published:** 2021-05-27

**Authors:** Martin Levine, Zsolt M. Lohinai

**Affiliations:** 1 Department of Biochemistry and Molecular Biology, University of Oklahoma Health Sciences Center, Oklahoma City, OK 73104, USA; 2 Department of Conservative Dentistry, Semmelweis University, H-1088 Budapest, Hungary; lohinai.zsolt@dent.semmelweis-univ.hu

**Keywords:** oral microbiome, gingival crevicular fluid, dentally attached cells, inflammation, lysine, cadaverine, lysine decarboxylase, butyrate, histone acetylation, sirtuin, stomach, nitric oxide

## Abstract

Periodontal disease is a common, bacterially mediated health problem worldwide. Mastication (chewing) repeatedly traumatizes the gingiva and periodontium, causing traces of inflammatory exudate, gingival crevicular fluid (GCF), to appear in crevices between the teeth and gingiva. Inadequate tooth cleaning causes a dentally adherent microbial biofilm composed of commensal salivary bacteria to appear around these crevices where many bacteria grow better on GCF than in saliva. We reported that lysine decarboxylase (Ldc) from *Eikenella corrodens* depletes the GCF of lysine by converting it to cadaverine and carbon dioxide. Lysine is an amino acid essential for the integrity and continuous renewal of dentally attached epithelium acting as a barrier to microbial products. Unless removed regularly by oral hygiene, bacterial products invade the lysine-deprived dental attachment where they stimulate inflammation that enhances GCF exudation. Cadaverine increases and supports the development of a butyrate-producing microbiome that utilizes the increased GCF substrates to slowly destroy the periodontium (dysbiosis). A long-standing paradox is that acid-induced Ldc and butyrate production support a commensal (probiotic) microbiome in the intestine. Here, we describe how the different physiologies of the respective tissues explain how the different Ldc and butyrate functions impact the progression and control of these two chronic diseases.

## 1. Introduction: Overview of Inflammatory Periodontal and Intestinal Diseases

### 1.1. Roles of Cadaverine and Butyrate

**Figure 1:**
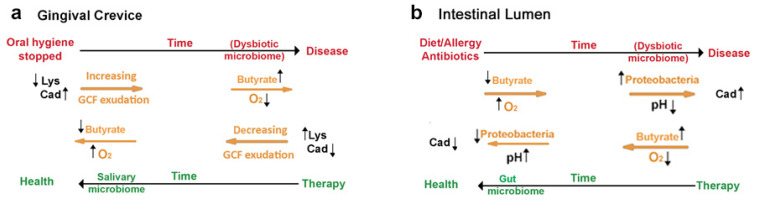
Summary: Cadaverine and butyrate production in periodontal and inflammatory bowel diseases. (**a**) *Restricting oral hygiene converts a commensal oral microbiome into a dysbiotic one*. Inadequate oral hygiene allows lysine decarboxylase from *E. corrodens* (LdcE) to deplete lysine from traces of an inflammatory exudate maintained by mastication, gingival crevicular fluid (GCF). GCF lysine is necessary to keep the dental epithelial attachment sealed from bacterial products, but LdcE action impairs this seal. Once impaired, the barrier cannot be re-established if oral hygiene remains inadequate, especially because GCF is a better bacterial substrate than saliva. Over time, oxygen access decreases, and the biofilm microbiome increases in mass and changes in composition. The bacteria hydrolyze GCF proteins and catabolize the amino acids to butyrate, which activates tissue destruction (periodontitis). Effective oral hygiene removes the butyrate, the dysbiotic biofilm, and the LdcE. The inflammation resolves and periodontal destruction stops. (**b**) *Various unrelated factors convert a commensal intestinal microbiome into a dysbiotic one*. Fatty diets, food allergies, autoimmune diseases, or excessive use of antibiotics irritate intestinal epithelial cell function and let oxygen into the lumen, causing a bloom of *Proteobacteria* that replace butyrate with lactate. The increased acidity increases bacterial production of cadaverine that protects the *Proteobacteria* from host leukocyte products. Treating the diet, allergic, or auto-immune diseases along with repeated intake of probiotic (butyrate-promoting) bacteria can remove the *Proteobacteria* and consequently the acidity and cadaverine content of the intestine. Original Figure.

The teeth lie in alveolar bone surrounded by the periodontium, which consists of the gingiva and periodontal membrane, both composed of many large bundles of fibrous collagen surrounded by small amounts of elastic (oxytalan) fibers and other connective tissue proteins. The outer surface of the gingiva in the oral cavity is covered with a layered epithelium that everts around each tooth to forms a thin crevice, at whose base is the dentogingival epithelial attachment. Bacteria from saliva adhere to teeth surfaces and extend into the crevice above this attachment. If the teeth remain uncleaned, they induce inflammation (gingivitis), a precursor of periodontitis in which the tooth attachments migrate apically until the teeth become loose and may eventually exfoliate [1]. We have discovered that gingivitis is initiated and maintained by lysine decarboxylase from *Eikenella corrodens* (LdcE), an enzyme that converts lysine to cadaverine and carbon dioxide [2]. Persistent gingivitis and cadaverine protect the biofilm microbiome and change its composition into one that synthesizes butyrate and mediates periodontitis [3,4,5]. A paradox is that the same combination of bacterial products, cadaverine and butyrate, maintain a commensal (probiotic) microbiome in the intestine. Figure 1 summarizes the roles and initiating factors of cadaverine and butyrate production in periodontal and intestinal diseases.

### 1.2. Progression of Gingivitis to Periodontitis

Periodontal disease affects 50% or more of the adult population aged 30 years or older [6] as the inflammation extends apically and destroys the periodontium [7]. The progression from gingivitis to periodontitis [1] occurs in bursts over many years [8], and the end result is tooth loss in older adults [9,10] in whom it may cause under-nutrition, as evidenced by low plasma albumin levels and low body mass index [11]. Some of the factors responsible for periodontal disease-mediated tooth loss were determined from dental insurance records in adults between 34 and 55 years of age who were retrospectively examined over 16 years [12]. Patients had no prior diagnosis of early periodontitis and were always offered professional preventive care to help maintain their oral hygiene. The primary association with tooth loss was the absence or presence of at least two visits annually for that care, but smoking, type 2 diabetes, or an interleukin-1 (IL-1) genotype [13] each contributed independently to significantly greater tooth loss [12,14]. ZL and his colleagues report that smoking constricts capillary vessels beneath the dentogingival attachment barrier [15], causing a reduced flow of gingival crevicular fluid (GCF) [16] that promotes periodontitis independently of diabetes and host IL-1 genotype. The association with diabetes is discussed in Section 4.

In Section 2, we describe the unusual nature of the dentogingival epithelial barrier (Section 2.1); the discovery of LdcE in dentogingival biofilms (Section 2.2); and the relationship of the overall structure of LdcE to that of the acid-inducible *Escherichia coli* lysine decarboxylase (LdcI) present in the intestinal microbiome (Section 2.3). Following a discussion of the composition of dental biofilms in periodontal health and disease (Section 2.4), we provide an in-depth discussion of how the production of cadaverine from lysine by LdcE impairs the dental epithelial barrier (Section 2.5), and how the inflammatory mediator, IL-1 [13], may alter the strength of the LdcE-induced innate immune response (Section 2.6). We then discuss how gingivitis generates a persistent inflammatory exudate that permits the outgrowth of a dysbiotic microbiome responsible for producing butyrate and periodontitis, as well as its control by oral hygiene (Section 2.7, Section 2.8, Section 2.9, Section 2.10 and Section 2.11).

### 1.3. Causes of Inflammatory Bowel Disease (IBD)

**Figure 2:**
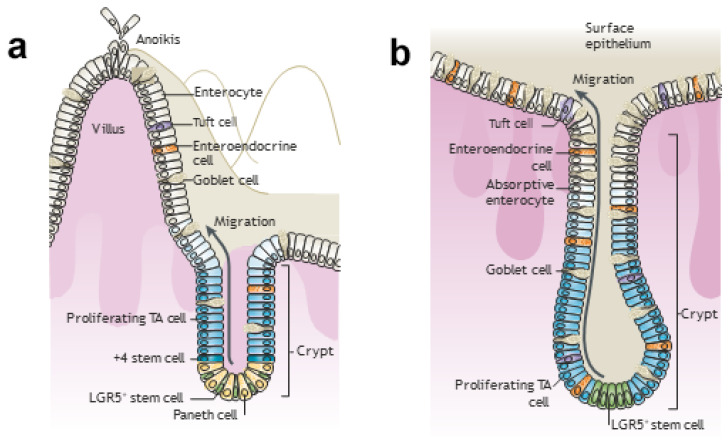
Illustrations showing the villus and crypt in the small and large intestines. (**a**) *Small intestine*. Epithelial cells of the small intestine are differentiated into nutrient-absorbing cells (enterocytes) and smaller amounts of other functioning cells [20]. These various cell types secrete mucins (goblet cells), antibacterial proteins (Paneth cells), a spectrum of biological effectors (tuft cells) [21], and gastrointestinal hormones (enteroendocrine cells). Two additional cell types, microfold cells responsible for mucosal (secretory) immunity and cup cells of unknown function, are not shown. As enterocytes (shown in white) shed, there is a reorganization of tight junction and adherens junction proteins. The former control the movement of ions and solutes between cells; the latter initiate and maintain cell-cell contacts [22]. Ejection is initiated by a procedure called anoikis, apoptosis coordinated with a signal for the adjacent enterocyte monolayer to plug the gap [23] (see top left and Section 3.2). In the crypts, LGR5+ (Leu-rich repeat-containing G protein-coupled receptor 5-expressing) stem cells (yellow) are intercalated with Paneth cells (light orange) at the crypt base (bottom right). Paneth cells contain antimicrobial peptides and immunomodulating proteins that regulate the composition of the intestinal flora. LGR5+ cells continuously generate rapidly proliferating transit-amplifying (TA) cells that occupy the remainder of a crypt and differentiate into the various functional cells listed above to replace those lost by anoikis at the villus tip. The +4 “reserve” stem cells just above the crypt base (darker blue than the proliferating TA cells) can restore LGR5+ CBC stem cells following injury. (**b**) *Large intestine (colon)*. LGR5+ stem cells at the crypt base generate rapidly proliferating TA cells in the lower half of the crypt (middle panel). TA cells subsequently differentiate into the same mature lineages as the small intestine epithelium (goblet cells; enteroendocrine cells; tuft cells; and absorptive enterocytes, now generally called colonocytes). They shed from the flat surface of the colon, and complete turnover occurs every 5–7 days. Reprinted with permission from [24]. Copyright 1986 John Wiley & Sons, Inc.

Food is digested by enzymes active in strong acid in the stomach and is passed into the duodenum where the acid is neutralized with a bicarbonate solution of proteolytic (digestive) enzymes secreted by the pancreas along with bile salts from the liver. Epithelial cells covering the interior (lumen) of the small intestine form a simple epithelium that differentiates into nutrient-absorbing cells (enterocytes) and smaller amounts of other functioning cells (Figure 2a). As digestion proceeds, smooth muscles beneath the epithelium push the intestinal contents along by squeezing and releasing the intestinal wall, known as peristalsis [17]. Most nutrients are absorbed in the distal portions of the small intestine, the jejunum, and ileum. Undigested substances enter the colon (large intestine), whose epithelial cells (colonocytes) reabsorb the water and bile salts. The epithelium of the colon possesses more accessory cells and fewer absorbing cells to control this increased load of bacteria compared with the small intestine (Figure 2b).

Chronic inflammation of the gastrointestinal tract is initiated by diet, food allergy, or antibiotics that results in an impaired epithelial barrier causing blood to enter the intestinal lumen [18]. It affects about 1.3% of the U.S. population, mostly as IBD, including Crohn’s disease, or ulcerative colitis. These conditions are frequently painful and often result in morbidity due to repeated hospitalization and surgical procedures [19]. In Section 3.1, we discuss the major composition of the intestinal microbiome and how intestinal inflammation can arise. In Section 3.2, we compare the factors that maintain the turnover of the intestinal attachment epithelium and compare them with factors that maintain the dentally attached epithelium (Section 3.3), as well as how the physiology of the intestines explains why cadaverine and butyrate are protective in the intestines. In Section 3.4, we consider why a bacterial or immunological association between IBD and chronic periodontitis has not yet been convincingly reported. In Section 4, we discuss therapy and prevention for both diseases, as well as factors in common with other inflammatory diseases. A central conclusion (Section 5) is that the loss of an epithelial barrier plays a key pathogenic role in both periodontitis and IBD, as well as the fact that oral hygiene in the oral cavity plays the same role as peristalsis in the intestines.

## 2. Ldc, Cadaverine, and Butyrate in Periodontal Health and Disease

### 2.1. The Healthy Dental Epithelial Attachment

**Figure 3:**
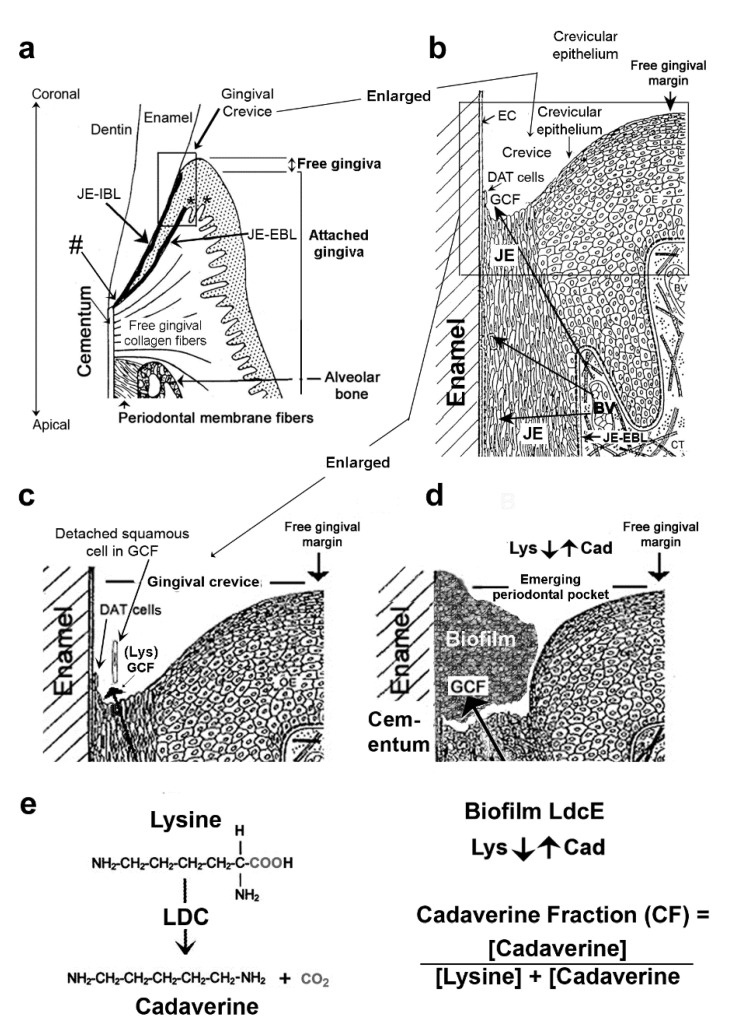
Structures of the gingiva and periodontium at various magnifications. (**a**) *Gingival region.* The gingival margin comprises the free and attached gingiva. The former forms the external wall of the gingival crevice and the latter is continuous with the outer oral epithelium. Junctional epithelial external and internal basal cell layers (JE-EBL and JE-IBL) meet at the cemento-enamel junction, a primary site of transit-amplifying cells for both basal cell layers (hashtag). Asterisks indicate 2 examples of where the stem cells lie relative to the JE (left) and an adjacent peg (see text, Section 2.1). Collagen fibers tightly anchor the attached gingiva to the alveolar bone. Figure 3a adapted with permission from [44]. Copyright 1990 Elsevier on behalf of CV Mosby. (**b**) *Gingival crevice.* The crevice (or sulcus) is bounded by enamel cuticle (EC), the coronal termination of dentally attached (DAT) cells producing the JE-IBL (shown in (**a**)), squamous JE cells, and crevicular epithelium. Arrows indicate the transudation route of fluid (GCF) through this epithelium from capillary blood vessels (BV) beneath the JE-EBL. GCF provides the constantly renewing DAT cells and their squamous cell progeny with nutrients [25,26]. (**c**) *Healthy gingival crevice.* The squamous cells bathed in GCF detach at the base of the crevice. Traces of biofilm derived from the salivary microbiota are present but not shown. (**d**) *Crevice deepening, an emerging periodontal pocket*. Biofilm derived from the salivary microbiome contains a lysine decarboxylase unique to *E. corrodens* (LdcE) that converts the lysine (Lys) from GCF to cadaverine (Cad). A greater cadaverine fraction therefore indicates greater LdcE activity and starvation of the DAT cells that stops their renewal (Section 2.5). Figure 3b–d adapted with permission from [45]. Copyright 1977 S. Karger AG. (**e**) *Lysine decarboxylation reaction mediated by LdcE*. LdcE enzyme activity was measured as the cadaverine fraction (CF) of the combined lysine plus cadaverine concentrations. CF = [Cadaverine]/([Lysine + Cadaverine]).

A healthy gingival crevice is bounded by tooth enamel on one side and the free gingiva (shown throughout all subsections of Figure 3) on the other. The coronal (upper) end of the crevice in Figure 3 is open to saliva, but its base is sealed by junctional epithelium (JE), also called the epithelial attachment [25]. The dentally and stromally attached cells of JE maintain a proliferative phenotype that renews the entire JE in about a week [25,26,27]. During this time, the older progeny differentiates into unattached squamous cells that make up the central portion of the JE, a region of loose intracellular junctions containing few desmosomes, as well as a few adherens and gap junctions. Continuous basal cell proliferation pushes the central squamous cells coronally into the gingival crevice from which they detach at the crevice base (Figure 3c).

Recently, Dutzan et al. reported that masticatory damage (from chewing or biting) activates interleukin-6 (IL-6) from gingival epithelial cells in germ-free mice [28]. Moreover, in humans, mechanical damage from occlusal forces leads to ‘‘secondary trauma from occlusion’’ [29] that enhances periodontal bone loss [30,31]. Related human studies also indicate that leukocytes are always present in the JE and GCF despite a clinically healthy gingiva and no biofilm [32,33], probably as a response to masticatory trauma. GCF therefore provides nutrients that enable the JE’s dentally attached (DAT) cells to maintain their dentogingival attachment, which acts as a barrier to bacteria. Figure 3b,c indicate the flow of GCF from underlying capillaries to the gingival crevice and oral cavity. Unfortunately, GCF provides better substrates than saliva for bacteria and likely stimulates the development of a microbiome responsible for dysbiosis in vivo [34]. If oral hygiene is inadequate, bacteria spread into the crevice (Section 2.5), where they encounter GCF. The bacteria remove lysine and starve the host-attached cells for this essential amino acid nutrient, thereby destroying the bacterial barrier (Figure 3d) and activating inflammation and periodontitis. This more complex, exclusively microbial mechanism gives the same result as chronic smoking (Section 1.2).

### 2.2. Discovery of Ldc and Butyrate in Dentogingival Biofilms

It is now many years since ML first proposed that dentally adherent biofilms contain water soluble substances that damage mammalian cells in vitro and cause periodontal inflammation and attachment loss in vivo [35]. Growth inhibiting or toxic substances were obtained by diluting pooled biofilm samples with sterile 75 mM sodium chloride. The mixture was Potter-Elvehjem homogenized and centrifuged, and the supernatant, called plaque (biofilm) extract, was sterilized by Millipore (Millipore-Sigma, Burlington, MA, USA) filtration [36]. The biofilm extracts inhibited the growth of various mammalian cell types [36,37] and contained a mixture of bacterial antigens [38,39], serum albumin from GCF, and amylase from saliva [40]. Passing the extract over Sephadex produced four toxic fractions [41,42], three heat-labile components, and a heat-stable component identified as a mixture of short chain fatty acids (SCFAs) within which butyrate was prominent [42,43]. The heat-labile components were all precipitated with ammonium sulfate, indicating a protein composition [41].

One toxic protein biofilm component was excluded from Sephadex G150 (Merck KGaA, Darmstadt, Germany); a second was 160 kDa; and the third was an unstable 60 kDa protein, likely a degradation product of the 160 kDa protein [41,42]. Polyclonal [40] and monoclonal antibody neutralization of cell culture growth [46] identified an 80 kDa antigen reactive to *E. corrodens* in biofilm extracts [47]. Subsequently, we identified that antigen is a monomer of LdcE [37] but with a sequence 65% identical to that of LdcI. Like LdcI, the active form of LdcE from biofilms is almost certainly a dimer [48], corresponding to the 160 kDa toxic unit. LdcE is toxic to mammalian cells in culture because it removes lysine from the culture medium by irreversible conversion to cadaverine and carbon dioxide. In the absence of lysine, an essential amino acid, cultured mammalian cells hydrolyze their own proteins for lysine until they die [37], a process called autophagic apoptosis [49]. As discussed in Section 2.5, lysine is a limiting nutrient for dentally attached epithelium at the base of the gingival crevice. Unlike LdcI, which is secreted extracellularly, LdcE is constitutive and remains on the *E. corrodens* cell surface, from which it can be removed by gentle homogenization [37].

### 2.3. Ldc Structure and Activity in Dentogingival and Intestinal Biofilms

The decarboxylation of lysine is the exclusive source of cadaverine in biology [50] and is present only in organisms that manufacture lysine de novo, such as bacteria, plants, and fungi. Cadaverine [51], along with volatile sulfur compounds [52], are the major contributors to malodor (halitosis) in the oral cavity. LdcE is optimally active at pH 5–10 [2], and therefore in the mildly alkaline environment of inflamed gingival crevices [53]. The structure of LdcI is complex. It is composed of five 160 kDa dimers that form two rings composed of half of each dimer to give a double, 5-membered ring in the crystallized active enzyme [54]. An essentially identical structure was recently confirmed for LdcE [55], implying that the LdcE dimer (160 kDa) and the large double ring, 800 kDa excluded from Sephadex G150, are active forms of this enzyme in dentogingival biofilms.

The only other bacteria thus far known to make Ldc in dentogingival biofilms are two *Capnocytophaga* species, *gingivalis* and *ochracea*, but only 10% of isolates of either species possess an Ldc compared with 80% of *E. corrodens* isolates [56]. Along with *E. corrodens*, *Capnocytophaga* species are early colonizers of biofilms in gingivally healthy adults [57], but the latter are essentially absent in the dysbiotic biofilms responsible for periodontitis, whereas the fraction of *E. corrodens* in healthy microbiomes and dysbiotic microbiomes is similar despite the latter possessing a thousand-fold more bacteria [5]. *E. corrodens* in saliva and dental biofilms accounts for about 60% of bite wound infections [58,59], and is a member of the *Hemophilus*: *Aggregatibacter* (previously *Actinobacillus*), *Cardiobacterium*, *Eikenella*, and *Kingella* (HACEK) group of fastidious bacteria associated with endocarditis [60]. As far as we can ascertain, it is not known whether LdcE contributes to *E. corrodens*-mediated bite infections and endocarditis.

### 2.4. The Commensal and Dysbiotic Dentogingival Biofilm Microbiome

Dentogingival biofilms extend into the gingival crevice containing 59 bacterial species associated with gingival health that are found in saliva. Yet, biofilms from periodontal pockets, deepened crevices caused by periodontitis, contain 88 other species rarely detectable in health. There are also 15 “core” bacterial species whose fraction of the microbiome remains the same in both health and disease [5]. Healthy species are mostly Gram-positive from the phyla *Firmicutes* (order *Lactobacillales*) and *Actinobacteria* (order *Actinomycetales*). If oral hygiene is inadequate, the order *Lactobacillales* from the phyla *Firmicutes* is increasingly replaced with Gram-negative anaerobes from the orders *Clostridiales* and *Veillonellaceae*, along with *Fusobacteria* (order *Fusobacteriaceae*), *Bacteroidetes* (order *Bacteroidales)*, and the phylum *Spirochaetes*. Different members of these oral phyla orders occur in the ileum and colon (Section 3.1).

The bacteria most strongly associated with periodontitis include a culture-based identification of a pathogenic triad of *Porphorymonas gingivalis* with *Tannerella forsythia*, both *Bacteroidetes* (order *Bacteroidales*), and *Treponema denticola* [61], but also many others including *Fusobacterium nucleatum* [5,62]. There is evidence that *P. gingivalis* infection is associated with Alzheimer’s disease and rheumatoid arthritis via the systemic blood supply [63,64], whereas oral *F. nucleatum* strains survive the stomach and may contribute to intestinal colorectal cancer [65], perhaps in association with cigarette smoking [66]. Unlike intestinal strains of *Fusobacteria*, oral strains are butyrate-producing, making their origin recognizable (Section 2.10), whereas the origin of *P. gingivalis* and other disease-related intestinal colonizers are less obvious [65]. Interestingly, *E. corrodens* is a betaproteobacterium, a class of the phylum *Proteobacteria*. *Proteobacteria* is a major phylum of Gram-negative bacteria that includes a variety of pathogenic genera, such as *Escherichia*, *Salmonella*, *Vibrio*, *Helicobacter*, *Yersinia*, and *Legionellales*. Many of these bacteria are important intestinal pathogens (Section 3.1) but rarely present in the oral cavity, where *E. corrodens* and various species of *Prevotella* most frequently account for *Proteobacteria* [5].

The change from a healthy biofilm derived from saliva to a dysbiotic one occurs initially within the gingival crevice, where GCF, the inflammatory exudate derived from plasma, replaces saliva [34]. The dentally adherent biofilm mass increases up to 1000-fold in association with periodontal pocket formation (Figure 3d) [5]. This large increase in bacteria is possible because of the much richer substrate environment of GCF and surrounding host proteins in the pocket compared a healthy crevice or saliva [34]. Dentogingival biofilm energy production changes from hydrolyzing glycans from the surface of salivary proteins [67] to hydrolyzing proteins in GCF [68]. The major catabolic end products in the biofilm change from a mixture of lactate, carbon dioxide, and water in health (micro-aerophilic) to acetate, propionate, and butyrate (strongly anaerobic) in disease. Cadaverine production is discussed in Section 2.5, Section 2.6, Section 2.7 and Section 2.8, and butyrate production in Section 2.9, Section 2.10 and Section 2.11.

As noted in Section 1.1, teeth surfaces provide a permanent solid surface for bacterial colonization. Their convex surfaces protect the gingival sulcus from being fully cleaned by solid foods such as apples [69]. Bacteria also adhere to other non-shedding, solid surfaces in the oral cavity, for example, orthodontic bands, bridges, or implants [70]. Although some dysbiotic bacteria may become detectable on oral mucosal surfaces [71], few biofilm bacteria attach to expelled squamous cells or leukocytes. Compared with the intestine, whose microbiome is attached to the epithelium by mucins and turns over as the mucins and epithelium turn over (Section 3.2), the dentogingival biofilm microbiome is stable and has to be controlled almost entirely by life-long oral hygiene.

### 2.5. LdcE Causes Gingivitis by Depleting Lysine, an Essential Amino Acid, by Conversion to Cadaverine

**Figure 4:**
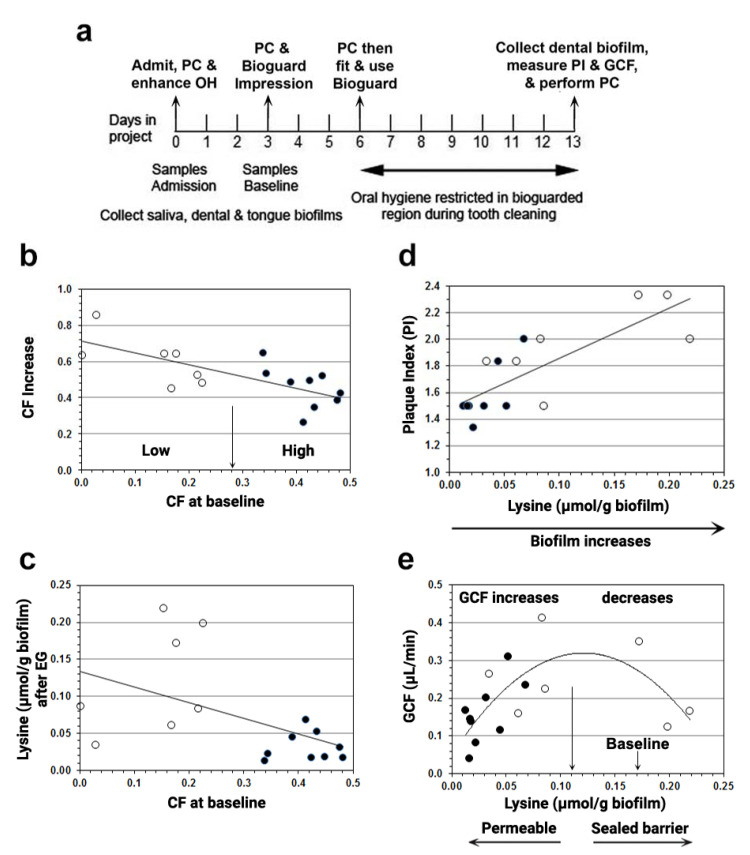
Associations of cadaverine fraction (CF) increase, biofilm lysine, biofilm accumulation, and GCF after oral hygiene were restricted for a week. (**a**) *Summary of methods*. PC = professional cleaning; OH = oral hygiene; PI = plaque index; GCF = gingival crevicular fluid. A bioguard splint was constructed to protect biofilm over an upper right or left quadrant and used only while tooth brushing during the week of EG. Note also that GCF was measured in the fluid fraction and therefore expressed per gram of biofilm (Section 2.5). (**b**) *Increase in cadaverine fraction (CF) is inversely related to baseline* CF. The central split (arrow) was determined by univariate clustering, which divided the participants into those with a small (○) or large (●) baseline CF. A large baseline CF gave a smaller CF increase. (**c**) *Biofilm lysine content is inversely related to baseline* CF. A large baseline CF resulted in less biofilm lysine (Mann-Whitney *p* < 0.01). (**d**) *PI associates with biofilm lysine content*. PI correlated with biofilm lysine (Lys) content after oral hygiene restriction (OHR). Spearman *R^2^* = 0.58, *p* < 0.001. (**e**) *GCF exudation rate regressed on biofilm lysine content after oral hygiene restriction.* Lysine depletion increases permeability moving left to the origin from the long vertical arrow near the center of the *x*-axis, i.e., at 0.11 µmol lysine/g biofilm, the minimal lysine concentration of normal blood plasma, 0.11 µmol/mL [78]. The mean lysine content before oral hygiene restriction (OHR, Table 1) to the right of the long vertical arrow is indicated by the short arrow. The quadratic relationship of GCF to lysine concentration after OHR was significant: least-squares regression, *R^2^* = 0.37, ANOVA *F* = 5.4, *p* < 0.02 (see text, Section 2.5). Adapted with permission from [73]. Copyright 2012 American Academy of Periodontology.

Experimental gingivitis (EG) is a procedure for studying the development of gingival inflammation in healthy young adults, usually for 3 weeks [72], but here only for 1 week [73]. Prior to starting EG, the teeth were thoroughly cleaned (Figure 4a), but once EG began, oral hygiene ceased, and salivary bacteria formed a biofilm often visible above the crevice within a day or two [74]. The saliva-originated biofilm extends into the crevice where bacterial antigens and salivary amylase mingle with albumin from GCF (Section 2.2). Because we had identified LdcE as depleting lysine in vitro (Section 2.2), we used capillary electrophoresis–laser-induced fluorescence, CE-LIF [75], to assay the lysine and cadaverine contents of biofilm and saliva before and after EG for a week, and compared them to plaque index (PI), a measure of biofilm accumulation [76], and to GCF exudation, a measure of inflammation [33,77].

Before starting EG, the lysine and cadaverine contents of dental biofilm were about equal, and almost 10-fold greater than in saliva or scrapings from the tongue surface, indicating that the source of biofilm lysine was GCF, not saliva [73]. After EG for a week, the biofilm lysine content fell and the cadaverine fraction increased (Table 1). In addition, Figure 4b shows that participants with a high baseline CF (filled circles) increased less after EG than low CF participants (unfilled circles). Figure 4c confirms that high-baseline CF participants had less lysine in their biofilm after EG because they converted more lysine to cadaverine than low CF participants. Taking CF as a measure of LdcE content, the results indicated that the LdcE content at baseline determines whether lysine decreases or increases after a week of EG. Indeed, other investigators [79] reported that the average increase in E. corrodens was 55% after a week of EG, similar to the mean percentage increase in CF in Table 1, further suggesting that the CF measures amount of LdcE, not activity [2].

We then examined whether baseline CF (LdcE content) also determines a participant’s response to biofilm accumulation and GCF exudation. Surprisingly, we found that the participants’ biofilm accumulation and GCF exudation depended on their lysine content, not their LdcE content. As a group, LdcE participants who were high at baseline had less biofilm, measured in Figure 4d as plaque index, PI [76], and less GCF exudation (filled circles) compared with low LdcE participants at baseline (unfilled circles). Biofilm lysine concentrations from high LdcE participants were also collinear with PI with respect to GCF after EG (Figure 4e), mimicking the classical relationship of gingivitis to biofilm accumulation [79]. Conversely, low LdcE participants at baseline exhibited more biofilm lysine and greater PI after EG, but no difference in GCF (unfilled circles in Figure 4d,e). Instead of continuing to rise with increasing lysine and PI, GCF plateaued and fell, giving a polynomial curve whose peak was centered around 0.11 µmol lysine/g biofilm (Figure 4e, long arrow).

It is noteworthy that 0.11 µmol lysine/g biofilm is equal to the minimal lysine concentration of healthy human plasma, 0.11 µmol lysine/mL [80]. This coincidence allows us to propose a lysine depletion-mediated pathomechanism of how gingival inflammation is initiated by LdcE de novo. Because the DAT cells must constantly renew in order to maintain their dentogingival attachment (Figure 3b), biofilm lysine must exceed 0.11 µmol/g dentogingival biofilm (legend to Figure 4e). After a week of EG, gingival inflammation (GCF) relates to PI (dentogingival biofilm accumulation) differently from the lysine concentration. Moving towards the left side of the curve peak, which is indicated by the long arrow in Figure 4e, the host’s DAT cells become increasingly lysine starved, proliferate less, lose attachment, and increase the permeability of the epithelial barrier to bacterial products. GCF, our measure of inflammation, also decreases because the lack of lysine decreases the availability of dentogingival biofilm, PI (Figure 4d).

Conversely, moving right from the arrow in Figure 4e, the lysine available to DAT cells exceeds the minimal amount and the DAT cell barrier becomes less penetrated by microbial-associated molecular patterns (MAMPs). Therefore, despite more stimulating biofilm to the right in Figure 4d,e, the biofilm MAMPs cannot invade the DAT cells and leukocyte-mediated innate immunity is reduced [81]. The results therefore indicate two groups that exhibit a minimal inflammatory response (GCF < 0.2 µL/min), five high LdcE baseline participants who were strongly depleted of lysine (<0.03 µmol/g biofilm), and two low LdcE baseline participants whose lysine content was ≥0.20 µmol/g biofilm.

### 2.6. Role of IL-1 in Our Lysine Depletion Mediated Pathomechanism of Gingival Inflammation

IL-1 is an innate (host) mediator of immune responses that activates cell- and antibody-acquired immunity to remove bacteria and facilitate healing [81]. IL-1 is encoded in the genome as two genes, IL-1α and IL-1β, whose respective amino acid sequences are only 34% homologous [82]. IL-1α is an alarmin that activates the initial response of the dentogingival attachment to the presence of MAMPs, as well as to released endogenous danger-associated molecular patterns, DAMPs [83]. Both MAMPs and DAMPs bind similar pattern recognition receptors (PRRs) [81,84]. One class of PRRs, Toll-like receptors (TLRs), are on the JE cell surface, and a second class, nucleotide oligomerization domain receptors (NLRs), are in the JE cytosol [84]. As noted in Section 2.1, mastication produces slight tooth movements that stress the JE DAT cells, producing DAMPs that upregulate IL-6 synthesis and secretion instead of IL-1α [81].

IL-1β expression requires IL-1α activation, and also the presence of an extracellular serine protease, granzyme B, for full activation [85]. During 3 weeks of EG, IL-1β expression catches up with IL-1α [86,87] and continues to increase in periodontitis [87]. Conversely, therapy and the re-institution of oral hygiene downregulates IL-1β expression as healing increases [88]. A lack of lysine (left side of Figure 4e) may activate the IL-1α response to DAMPs, and eventually also IL-1β if DAT cells become lysine-deprived [81]. Indeed, a predictive modeling study of gingivitis severity after 3 weeks of EG indicates that one-third of their participants exhibited little increase in gingival index and microbiome composition, unlike the remaining two-thirds [89]. The five participants who were exceptionally depleted of lysine by exceptionally strong LdcE activity in our study [73] displayed a weak innate immune response (GCF < 0.2 µL/min shown in Figure 4e), a similar fraction to that in a 3-week EG study reported by Huang et al. [89]. We are currently investigating the origin of the weak proinflammatory response by examining genetic variants of IL-6 and IL-1 in our study participants [12,28].

### 2.7. Lysine Degradation in Gingivitis and Periodontitis

Prior to therapy for periodontal disease, lysine degradation is over-represented in biofilms from periodontal pockets (Figure 3d), and lysine biosynthesis is significantly under-represented [90]. In another study, 9 of 22 bacterial genes involved in amino acid metabolism showed differences in abundance between periodontitis and healthy groups, and four genes, including a gene for Ldc, exhibited an increased abundance in periodontitis [91]. A third study indicated that after 2 weeks of EG, cadaverine in saliva was increased by 69% [92]. A fourth study used the periodontally inflamed surface area (PISA), a combination of clinical attachment level (CAL), and bleeding on probing (BOP) [93] to relate salivary metabolomics to periodontitis severity before and after debridement [94]. Multivariate analyses indicated that cadaverine in saliva was primarily associated with a higher PISA, whereas salivary metabolites that decreased after debridement included those associated with butyrate metabolism and lysine degradation [95]. These studies indicate that persistent gingival inflammation produces a dysbiotic biofilm that, together with activated host leukocytes, degrades proteins in the GCF and gingival tissues [96]. Many of the resulting amino acids are catabolized and reduced to butyrate, which is excreted along with lesser amounts of propionate and acetate (Section 2.10). Other dysbiotic biofilm bacteria such as *E. corrodens* grow by oxidizing amino acids but reduce nitrate to nitrite instead of producing SCFAs.

### 2.8. Cadaverine Protects Bacteria from Reactive Nitrogen and Oxygen Species in Periodontal Disease

In many chronic inflammatory diseases such as periodontal disease [97,98] and IBD [99], the interaction of phagocytic cells with invading pathogens results in nitric oxide (NO) formation from a family of isoenzymes called inducible NO synthases (iNOS) that convert arginine to citrulline and NO [100]. In the gastrointestinal tract [101], NO can also be produced by bacterial nitrite reductase and spontaneously from nitrite in an acidic environment [102]. Once formed, NO interacts with a superoxide anion to form peroxynitrite, the most stable of many reactive nitrogen species (RNS) obtained from NO. Peroxynitrite is responsible for bactericidal activity, but it also interacts with degraded bacterial cell wall fragments. This latter interaction triggers a positive feedback loop resulting in greater iNOS induction and greater amounts of other reactive nitrogen species as well as more peroxynitrite [103]. Besides the acidic environment of the stomach inducing LdcI from *E. coli* and other enterobacteria (Section 3.1), the colon is also slightly acidic and also induces LdcI from these bacteria. Cadaverine protects *E. coli* by closing its porins not only to acid penetration but also to RNS [104,105] and reactive oxygen species (ROS) in the colon [106].

The production of cadaverine by *E. corrodens* in the alkaline environment of inflamed crevices and periodontal pockets [53] protects the oral biofilm microbiome from the bactericidal and bacteriostatic effects of RNS and ROS. Cadaverine is a dibasic amine that alkalinizes the environment of the gingival crevice [51] and maintains native bacterial protein structures by preventing tyrosine residue nitration [107]. The cadaverine produced by LdcE therefore promotes periodontitis by protecting the dysbiotic microbiome from these host leukocyte mediators [31]. *E. corrodens* comprises the same percentage of both healthy and dysbiotic microbiomes [5], and it grows by oxidizing a few amino acids (proline, glutamate, and serine) to ketoacids and ammonia in exchange for reducing nitrate to nitrite [108]. Once gingival inflammation develops, the increase in GCF provides a better source of amino acids and nitrate for the growth of *E. corrodens* and other dysbiotic bacteria [5], all of which are protected from host ROS and RNS by cadaverine. In the colon, cadaverine uses the same mechanisms, but normally acts to preserve a commensal (probiotic) microbiome (Section 3.1).

### 2.9. Butyrate Induces Periodontitis

As introduced in Section 2.7 and Section 2.8, lysine degradation to cadaverine ultimately promotes bacterial butyrate production by both enhancing GCF exudation and stabilizing the resulting dysbiotic microbiome [73]. Butyrate appears in GCF at concentrations of 0.3–16.0 mM, with the smallest amounts in mildly inflamed crevices [4] and greatest amounts in deep pockets [109,110]. In vitro studies indicate that 0.5 mM butyrate inhibits the growth of most types of cultured mammalian cells by 50% [111] and completely at 5.0 mM [111,112,113]. Other studies indicate that butyrate at 4–10 mM produces peroxides and other ROS that cause cell cycle arrest or apoptosis in cultured human gingival fibroblasts [114], apoptosis in T lymphocytes [113], and either necrosis [111] or apoptosis-mediated cell death in epithelial cells [115].

The growth inhibiting and necrotic effects of butyrate on epithelial cells may explain why the JE degenerates in periodontitis [116]. Butyrate activates high mobility group box 1 (HMGB1), an abundant architectural chromatin-binding protein in the nucleus, and moves it to the cytosol where it acts as a DAMP (Section 2.6) [83]. High concentrations of butyrate (>10 mM) induce human epithelial cells to release HMGB1 extracellularly in vitro, and HMGB1 expression is increased in GCF from periodontal pockets compared with healthy crevices in vivo [109]. Immunohistochemical staining reveals that HMGB1 is exclusively dislocated from the nucleus to the cytoplasm in periodontal pocket epithelium, but not in uninflamed gingival epithelium [117]. Interestingly, exposing the human gingival epithelial cell line Ca9-22 to 5 mM butyrate releases HMGB1, even when inhibitors of apoptosis are present [109]. This indicates that HMGB1 releases signals that cause necrosis [118], especially if the epithelial cells are already impaired prior to butyrate exposure [119].

### 2.10. Butyrate-Producing Bacterial Pathways

**Figure 5:**
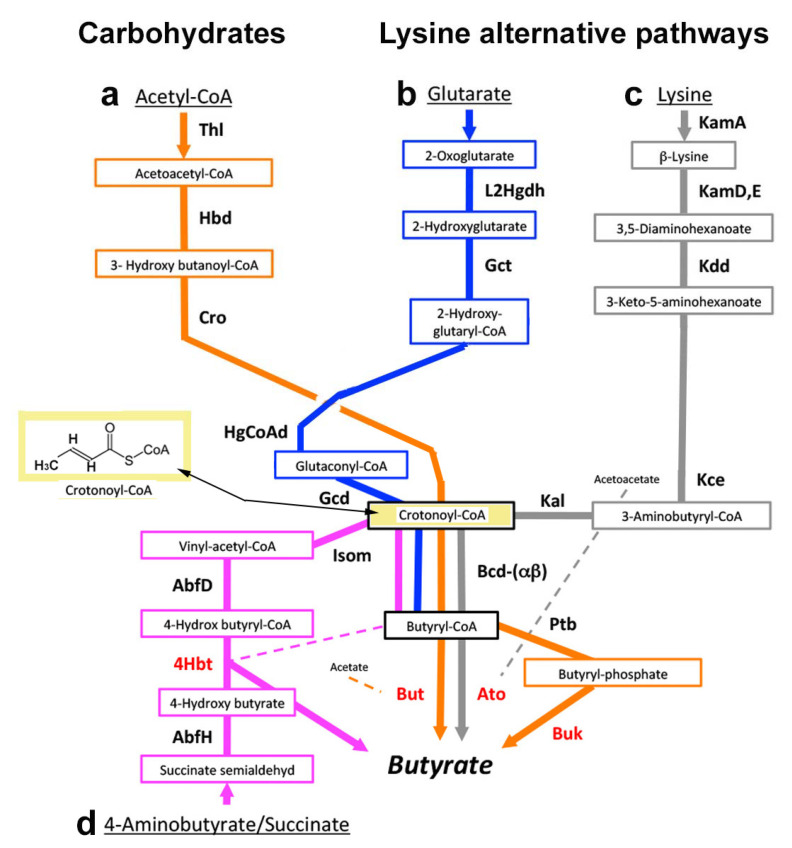
Anaerobic pathways for butyrate synthesis. Major substrates are carbohydrates and the amino acids lysine and glutamate. The common pathway: crotonyl-CoA (yellow rectangle) is common to all 4 pathways. In most bacteria, crotonyl-CoA is metabolized by the terminal genes (red) to butyryl-CoA by butyryl-CoA dehydrogenase (Bcd) acting in reverse, and then to butyrate by butyryl-CoA/acetyl-CoA transferase (But). In a few bacteria, butyryl-CoA is first phosphorylated by butyrate phosphate butyryltransferase (Ptb) and then dephosphorylated to butyrate with butyrate kinase (Buk). (**a**) *Acetyl-CoA (orange arrows*): product of carbohydrate metabolism. Acetyl-CoA is converted to crotonyl-CoA using three enzymes: thiolase (Thl), hydroxybutyryl-dehydrogenase (Hbd), and crotonase/enoyl-CoA hydratase (Cro). (**b**) *Glutarate (blue arrows): product of lysine or tryptophan metabolism* [121]. Glutarate is converted to crotonyl-CoA by 2-hydroxyglutaratedehydrogenase (L2Hgdh) followed by glutaconate-CoA transferase (Gct), 2-hydroxyglutaryl-CoA dehydrogenase (HgCoAd), and glutaconyl-CoA decarboxylase (Gcd). (**c**) *Lysine (gray arrows): unique path*; lysine is metabolized to crotonyl-CoA by lysine 2,3-aminomutase (KamA), lysine 5,6-aminomutase (KamD), 3,5-diaminohexanoate dehydrogenase (Kdd), 3-keto-5-aminohexanoate cleavage enzymes (Kce), and 3-aminobutyryl-CoA ammonia lyase (Kal). In a few bacteria, acetoacetate may also be converted to butyrate using the enzyme butyryl-CoA acetoacetate-CoA transferase (Ato). (**d**) *4-Aminobutyrate/succinate* (purple arrows from the bottom of the figure): 4-aminobutyrate (GABA) is a product of glutamine [122], obtained by glutaminase removing the glutamine amide group to form glutamate. The glutamate amino group is removed by glutamate dehydrogenase to form succinate-semialdehyde and converted to crotonyl-CoA by 4-hydroxybutyrate-dehydrogenase (AbfH), butyryl-CoA-4-hydroxybutyrate-CoA transferase (4Hbt), and 4-hydroxybutyryl-dehydratase (AbfD). AbfD also possesses vinyl-acetyl-CoA isomerase activity (Isom). Not shown are how aspartate, arginine, and putrescine are converted to butyrate by the 4-aminobutyrate/succinate path. Aspartate is converted to succinate semi-aldehyde by three enzymes, namely, aspartate aminotransferase, fumarate transferase, and acyl-CoA transferase. Arginine is decarboxylated to agmatine, which is hydrolyzed to urea and putrescine. The putrescine reacts with ketoglutarate to produce 4-aminobutyraldehyde, which is oxidized to 4-aminobutyrate and metabolized like glutamine [123]. The common step of all four pathways is the synthesis of crotonyl-CoA, which precedes butyryl-CoA and butyrate. Adapted from [120].

There are four butyrate production pathways: from acetyl-CoA, glutarate, lysine, and 4-aminobutyrate [119,120] (Figure 5). The healthy gut microbiome metabolizes complex carbohydrates to pyruvate, which is oxidized to acetyl-CoA. Two molecules of acetyl-CoA condense to form acetoacetyl-CoA, which is reduced to butyrate (Figure 5a) instead of pyruvate to lactate. Three other bacterial pathways also produce butyrate. Bacteria that reduce lysine and tryptophan to butyrate utilize the glutarate path (Figure 5b), but other bacteria can produce butyrate exclusively from lysine by a separate, unique path (Figure 5c) and therefore deprive the crevice of lysine without producing cadaverine. Bacteria that utilize glutamine, aspartate, arginine, and putrescine all produce butyrate using the 4-aminobutyrate/succinate path, shown at the bottom of Figure 5d. All four pathways terminate in crotonyl-CoA, which is converted to butyryl-CoA, and then to butyrate by butyryl-CoA/acetyl-CoA transferase (But) in most butyrate-synthesizing bacteria. On the other hand, a few of these bacteria instead produce butyrate by phosphorylating butyryl-CoA and dephosphorylating it to butyrate (Figure 5).

In the gingival crevice, *Fusobacterium nucleatum* ATCC 25586 can adhere to and invade human oral epithelial cells where it causes periodontitis or abscesses [124]. Other oral strains of *F. nucleatum* may cause invasive infections of the head, neck, lung, liver, heart, or brain, or pass through the umbilical cord and cause preterm birth, stillbirth, and neonatal sepsis [124]. Alternatively, they may spread from the oral cavity to colonize the intestine in association with appendicitis, IBD, or colonorectal cancer [65]. Intestinal strains of *F. nucleatum* cannot produce butyrate because the gene for *But* is absent, whereas in oral strains, this gene is present but flanked by a transposase and is well separated from the DNA-encoding region of the other butyrate production genes [119]. Nevertheless, the extent to which diseases caused by oral strains of *Fusobacteria* are a consequence of their butyrate production remains uncertain [125] (see also Section 2.4 and Section 3.1).

*F. nucleatum* can also produce butyrate by using the lysine pathway in association with “aggressive” periodontitis [62], redefined in 2017 as a generalized, rapidly progressive periodontitis [126]. The lysine path exists in other major periodontopathogens, such as *Filifactor alocis* or *Porphorymonas gingivalis* [127,128]. Nevertheless, butyrate is more commonly produced by the 4-aminobutyrate/succinate path (Figure 5d) by these and other bacteria such as *Tannerella forsythia* [127,129,130,131]. Primary use of the lysine path by oral bacteria may be a rare cause of lysine depletion independently of the LdcE path and may associate with the equally uncommon generalized, rapidly progressive periodontitis [62].

### 2.11. Cadaverine and Butyrate after Periodontal Therapy

Established therapy for periodontal disease consists of mechanical debridement to remove calcified and soft bacterial biofilm deposits, a procedure called scaling and root cleaning (SRP), followed by twice-daily intensive oral hygiene. Most patients respond to debridement and self-administered oral hygiene without antibiotics, but a minority respond poorly (“refractory”). In a study of patients who had lost teeth because of severe periodontitis, 60% were smokers and 40% were non-smokers [132]. Because refractory non-smokers are difficult to identify, there is a tendency to over-prescribe antibiotics, for example, systemic amoxicillin (500 mg thrice daily) and metronidazole (250 mg thrice daily) for 7 to 14 days after the first debridement visit [133]. Antibiotics may clear slow-resolving inflammation in non-smoker patients, but recent reports indicate that tooth attachment is regained more after conventional debridement without antibiotics. Furthermore, the frequent use of antibiotics for periodontitis is undesirable because it promotes the spread of bacterial resistance [134,135].

We examined biofilm lysine and cadaverine contents following therapy and compared them to the results of our EG study depicted in Table 1 (Section 2.5). Following a 1-year program of carefully monitored debridement without antibiotics, lysine and cadaverine levels in seven individuals who lost periodontal attachment, poor responders (PR) were compared with 9 good responders (GR), 6 healthy volunteers (HV), and 33 untreated patients presenting with clinical evidence of gingivitis or mild periodontitis (Table 2). In the seven poor responders, the biofilm lysine concentration after therapy was the same as in 16 healthy individuals after EG (Section 2.5), whereas the lysine concentration of the good responders resembled that of the healthy volunteers [3] and the healthy individuals before EG (Table 1). Yet, the cadaverine plus lysine concentrations in poor responders were only about 40% of that in participants before or after EG, or in good responders or healthy volunteers (Table 2).

The loss of lysine in poor responders to therapy was therefore not balanced by a greater cadaverine content. One possibility is that the “missing” lysine was converted to butyrate, as reported for “aggressive” periodontitis [62] (see Section 2.10). A surprise was that untreated patients exhibited an exceptionally low cadaverine fraction despite their lysine content resembling that of healthy EG volunteers and good responders (Table 2, far right column). Extensive proteolysis by host leukocytes and the microbiome may increase the fraction of lysine available to LdcE (Section 2.4), whereas debridement likely slows proteolysis and decreases lysine, allowing LdcE to increase CF to that of the healthy and successfully treated groups in Table 1 and Table 2.

As introduced in Section 1.2., smokers are three times more likely to develop periodontal disease than non-smokers [136], and often respond poorly to therapy [137,138]. Correspondingly, among four of the five poor responder smokers who provided enough dentogingival biofilm for total amino acid assay, all amino acids were depleted, such as lysine, whereas lysine alone was depleted in dentogingival biofilm provided by one of the two poor responder non-smokers who provided enough dentogingival biofilm for total amino acid assay [3]. Stopping smoking is essential for periodontal therapy to be successful, and only non-smoking patients responding poorly should receive adjunctive antibiotic therapy to remove bacteria making too much butyrate from lysine. On the basis of these observations, we conclude that low lysine plus cadaverine levels in dentogingival biofilm from non-smokers within a few months of starting therapy may be a biomarker that indicates a need for antibiotics to eliminate the conversion of lysine to butyrate in these patients [3] (Section 2.10).

## 3. Ldc, Cadaverine, and Butyrate in Intestinal Health and Disease

### 3.1. The Intestinal Microbiome, Cadaverine, and the Intestinal Epithelial Barrier

The stomach is acidic and relatively sterile, although bacterial cadaverine production allows some bacteria to survive and reach the ileum, the distal end of the small intestine (Section 1.3). There, the bacteria increase to approximately 10^8^ and 10^12^ bacteria per gram dry weight of ileal contents and colonic contents, respectively [17]. In health, most of these bacteria are of the families *Lachnospiraceae* and *Ruminococcaceae*, members of the phylum *Firmicutes* of the orders *Clostridiales* and *Clostridia*, respectively. The remainder are *Bifidobacteria*, members of the phylum *Actinobacteria*, order *Bifidobacteriales*. These latter bacteria are all strict anaerobes that ferment dietary fiber (non-digestible complex carbohydrates) to butyrate instead of lactate [139,140].

**Figure 6:**
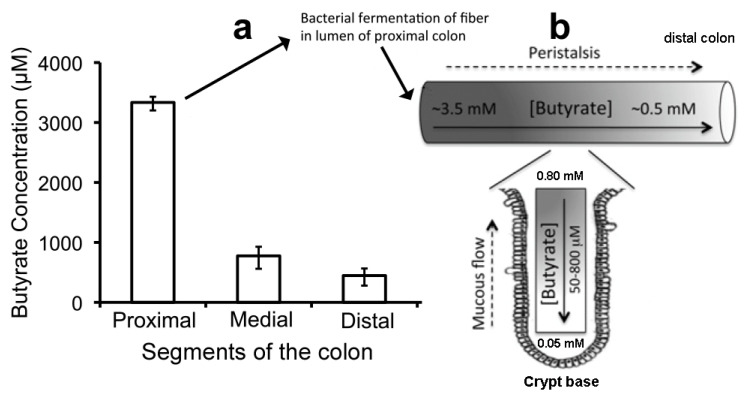
Peristalsis and butyrate concentration in the intestinal lumen. (**a**) *Butyrate levels in the lumen of proximal, medial, and distal segments of mouse colon.* (**b**) *Model showing two butyrate gradients in the mammalian colon*. The proximal-to-distal luminal gradient arises because most bacterial fermentation occurs in the proximal colon, and butyrate that is not absorbed by proximal colonocytes is pushed distally with luminal contents due to peristalsis. The luminal-to-crypt gradient also arises from peristalsis squeezing an upward flow of mucous secreted continuously into the crypt by goblet cells. Adapted with permission from [156]. Copyright 2012 Elsevier.

A fourth phylum, *Bacteroidetes*, is represented by additional strict anaerobes that preferentially produce acetate and propionate from dietary fiber [141,142]. These bacteria colonize the distal colon and cause the molar ratio of butyrate to acetate plus propionate to fall from 20% in the proximal colon to 10% in the distal colon [143]. The concentration of butyrate is 3.5 mM in the proximal colon (Figure 6a) but only 0.5 mM in the distal colon due to the greater predominance of *Bacteroidetes*. SCFAs make the colon slightly acidic, pH 6.5 [141], and many commensal bacteria possess an LdcI that is acid-induced in both stomach and colon to make cadaverine, which enhances bacterial survival in the gastrointestinal tract [104] (Section 2.8). Furthermore, unlike saliva in the oral cavity, where lysine is virtually absent and only available from GCF, lysine should be plentiful from the digestion of proteins throughout the intestinal tract (Section 1.3).

Diets that are overly rich in fat promote allergies and impair the epithelial barrier [144] by altering the colonic microbiome (dysbiosis), or by preventing colonocytes from absorbing or metabolizing butyrate [18,145]. In addition, the lack of stomach acid induced by drugs such as proton inhibitors could allow bacteria from food or a periodontally diseased oral cavity to cause colonorectal cancer (Section 2.4 and Section 2.10). Colonocytes stressed by these changes activate a strong proinflammatory agent, tumor necrosis factor-α (TNFα) [146]. More importantly, the stressed colonocytes leak blood and oxygen into the lumen along with the iNOS produced by leukocytes attracted to the region and activated by TNFα [147]. The presence of cadaverine supports the growth of *Proteobacteria*, a phylum composed of many Gram-negative bacteria enterotoxin-producing pathogenic *Salmonella*, *Vibrio*, *Shigella*, and *Escherichia*, facultative anaerobes that grow anaerobically by reducing nitrate to nitrite such as *E. corrodens* [148]. *E. corrodens* is a betaproteobacterium, and *E. coli* is a gammaproteobacterium. Within these bacteria genera, those possessing LdcI do not possess the enterotoxin and vice versa, apparently because too much cadaverine prevents their expression of an adhesin that attaches these bacteria to the intestinal epithelium where the enterotoxin is active [149].

The NO from bacterial nitrite or host iNOS combines with oxygen from the blood to form peroxynitrite and other reactive nitrogen species in the mild acidic environment of the colon [150] Critically, the presence of proteobacteria changes the fermentation of dietary fiber from butyrate to lactate, which is more acidic than SCFAs. The greater acidity of the colon enhances the spontaneous conversion of nitrite to peroxynitrite, and also the production of diamines, cadaverine, and putrescine [151], which protect the dysbiotic microbiome (see Section 2.8). Nevertheless, enough reactive nitrogen species is now free to further impair the host intestinal epithelium and its underlying stroma. Inflammation in the intestines therefore contributes to a bloom of *Proteobacteria* and reactive nitrogen species, providing a plausible explanation for how difficult it is to treat the impaired epithelial barrier in IBD.

### 3.2. Butyrate Maintains Epithelial Turnover for a Healthy Intestinal Epithelial Barrier

The intestinal lumen is covered with a simple epithelium, a column of stromally attached cells derived from endoderm. Stem cells at the base of the crypts of Lieberkühn (Figure 6) produce 14 to 21 transient amplifying cells every hour [152], leading to complete intestinal epithelial renewal (turnover) within a week, akin to the JE [25] (Section 2.1). This turnover ensures that only the most metabolically able cells perform the functions discussed in Section 1.3 and illustrated in Figure 2 [20,146]. However, epithelial cell removal is engineered by a detachment-induced modified apoptosis called anoikis [23,146,153]. Anoikis occurs at the tip of each villus in the small intestine and within the flat surface of the colon. The barrier and absorption properties of intestinal epithelium are maintained [153], but whether extrusion is initiated by exposure to more butyrate in the lumen or by unknown signals from the younger surrounding cells is not clear [146]. In the small intestine, large amounts of mucus from goblet cells (Figure 6) form a diffusion barrier that limits bacterial penetration into the epithelium. In the colon, an epithelial-attached inner mucus layer is almost bacteria-free. When old epithelial cells are shed, they enter the mucous flow, which carries a mixture of bacteria non-metabolized butyrate and undigested food to the rectum to be excreted as feces. This process is more complex than that of JE (Section 2.1). The microbiome of dentogingival biofilms is tightly attached to non-shedding teeth surfaces (Section 2.4) unless removed by SRP (Section 2.11), whereas the intestinal epithelial lumen is continually cleaned by peristalsis causing a flow of mucous towards the rectum for excretion [154].

### 3.3. Butyrate Paradox Resolved

The epithelial lining of the intestinal lumen primarily absorbs and metabolizes butyrate, but also some propionate and acetate, by transferring oxygen from the underlying capillary plexus to mitochondria where all of these SCFAs are oxidized. In the ileum, enterocytes use the ATP produced from catabolizing SCFA to absorb the products of digestion, and in the colon, colonocytes use that energy to re-absorb large amounts of water and salt (Section 1.3). Butyrate is an especially important nutrient for enterocytes and colonocytes [155]. Once in these cells, it is oxidized to acetyl-CoA and enters the Krebs cycle and is metabolized to ATP in mitochondria. Acetyl-CoA is a cofactor for histone acetyltransferases (HATs) that upregulate a set of target genes favoring cell proliferation [156]. There are also histone deacetylases (HDACs) that inhibit cell proliferation and help control cancers (Section 4.2). Most of the butyrate produced by bacteria in the colon lumen accumulates within the feces and is excreted [157]; only 0.002–0.004 mM butyrate reaches the systemic circulation [158].

Butyrate has opposing effects on the growth of normal versus cancerous colonocytes, a paradox that has been poorly understood [156]. For example, 8mM butyrate caused potent cytotoxicity in the murine normal colonic epithelial cell line MCE301 after growth for 48 h [159], whereas the human colorectal carcinoma Caco-2 cells required 20 mM butyrate. Cells that proliferate, especially cancer cells, use glucose to produce energy (ATP) for new growth, a well-established preference called the Warburg effect [160]. Thus, when the Warburg effect is blocked, the resistance of cancer cells to butyrate is blocked, and they are killed at the same levels as normal colonocytes. As a result, large amounts of butyrate are not readily metabolized by cells that are constantly turning over such as intestinal epithelium or the JE dental attachment. Such cells accumulate butyrate in the nucleus to 0.5 mM, causing the butyrate to act as a histone deacetylase inhibitor (HDACi). This action upregulates genes such as HMGB1, which promotes cell death and necrosis in vitro [156] (Section 2.9). In normal cells, very low levels of butyrate mediate histone acylation and deacylation (HAT and HDAC) that activate and control growth, whereas the inhibition of histone deacetylation (HDACi) activates inflammation and cell death.

How, then, can intestinal epithelial cells possibly grow on butyrate? The answer is found in a groundbreaking detailed investigation published by Donohoe et al. [156]. These authors explain why and how the concentration of butyrate available to the intestinal epithelium is controlled by mucous and peristalsis. Peristalsis moves food along the digestive tract so that its nutrients can be absorbed, as well as so indigestible foods are excreted. Mucous lubricates the intestinal epithelial surface, which not only facilitates peristalsis but also substantially reduces the butyrate concentration at colonocyte surfaces, especially at the base of the crypts. In their study of butyrate concentrations in the mouse colon lumen, liquid chromatography–tandem mass spectrometry detected butyrate at 3.5, 0.8, and 0.5 mM, in the proximal, medial, and distal regions of a peristaltic crypt, respectively (Figure 6a) [156]. Moreover, the butyrate concentration exposed to the epithelial cell surface at the base of a crypt was only about 0.05 mM [155,156,161], small enough utilize the acetyl-CoA/HAT mechanism and stimulate colonocyte proliferation (Figure 6b). As the cells move up from the crypt base, they move to the lumen where even mucous cannot protect them from the 10–70-fold greater butyrate concentration that facilitates HDACi release of HMGB1 and anoikis (Figure 2 and Section 3.1).

On the other hand, there is no such physiological control of the butyrate concentration in periodontal disease. Pockets resembling crypts form around the teeth, but there is no movement other than professional scaling and oral hygiene to remove bacteria and butyrate from teeth surfaces in a pocket. As noted in the 3rd paragraph of Section 2.4., GCF exudation promotes intense, dysbiotic bacterial growth, and is therefore ineffective as a washing mechanism. Instead, there is a slow increase in butyrate concentration, and therefore in deacetylase inhibition (HDACi) and activation of HMGB1 (Section 2.9). As a consequence, butyrate destroys the periodontium by persistently activating inflammation and cell death. Most of the actual destruction is endogenous but mediated by increasing levels of butyrate as the pocket keeps deepening [4].

### 3.4. Immunological Association of IBD with Periodontal Disease

A meta-analysis of published cross-sectional studies recently reported that patients with IBD have a greater risk of periodontitis compared to non-IBD patients [162]. A recent study conducted on mice indicated that oral and gastrointestinal mucosal microbiomes are directly connected microbiologically and indirectly connected immunologically [163]. Periodontitis developed quickly after ligatures placed on the teeth trapped large numbers of *Klebsiella* and *Enterobacter* species (gammaproteobacteria) due to the mice continually ingesting feces where these species are common [164]. As many of the same bacteria colonized both oral cavity and gut in this study, it is not surprising that immune crosstalk between the oral cavity and gut was detected. The major problem is relevance to human periodontitis. Human dentogingival biofilms rarely contain gammaproteobacteria other than *E. corrodens* (Section 2.4), unlike the human intestines (Section 3.1), and high-throughput 16S rRNA gene sequencing indicates that both *Klebsiella* and *Enterobacteria* are absent from the list of 88 periodontitis-associated species [12] (Section 2.4).

Some periodontal pathogens such as *F. nucleatum* (Section 2.10) and *P. gingivalis* [165,166] can indeed translocate to the human gut, but we are not thus far aware that any human periodontal pathogen can change the human colon microbiome sufficiently so as to activate oral T17 cells that might also translocate to the gut [163]. A specific causality link between IBD and human periodontal disease therefore remains to be established, and a simpler possibility is that affected patients develop periodontitis by their IBD morbidity preventing them from maintaining proper oral hygiene. The question is therefore whether optimal periodontal care can treat IBD. Longitudinal studies should be designed to investigate whether adults with moderate periodontitis are more likely to develop IBD. One possibility is to determine what proportion of individuals aged 35–45 with moderate periodontitis in the National Health and Nutrition Examination Survey (NHANES) developed IBD later in life compared with those already diagnosed with IBD.

## 4. Implications for Periodontal and Intestinal Diseases: Therapy, Prevention, and Links to Other Diseases

### 4.1. Periodontal Disease

DAT cells utilize the lysine from traces of GCF induced by mild tooth movement during mastication (Section 2.3). Restricting oral hygiene brings LdcE into contact with the GCF, and the resulting depletion of lysine impairs the JE’s DAT cell attachment barrier to bacteria. An innate immune response develops and the enhanced GCF exudation promotes dysbiotic changes in the dentogingival biofilm unless oral hygiene is re-established within a day or two [34]. The simplest possibility is to replenish the lost lysine with a lysine mouthwash, but lysine activates LdcE in vitro [37] and probably also in saliva where the additional cadaverine would cause halitosis (Section 2.3). Another approach is to use a lysine analog such as DL-difluoromethyl-lysine or tranexamic acid, both of which inhibit LdcE. However, the former had poor activity, and tranexamic acid retarded both GCF exudation and its lysine content [2]. Neither of these approaches seem promising.

Antibodies to LdcE inhibit both LdcE activity and gingivitis in beagle dogs for about 6 weeks [167], but repeated immunization might inhibit LdcI and upset the intestinal microbiome. Interestingly, the amino acid sequence of the *N*-terminal region of LdcE is unique and is absent from other oral and intestinal bacterial Ldcs. It may therefore be possible to restrict the antibody specificity to the N-terminal region of LdcE, which should not affect the activity of LdcI. Yet, another possibility is to develop probiotics [168]. Probiotics work by bacteria and yeasts producing substances such as bacteriocins that might eliminate *E. corrodens* from the oral microbiome and prevent its appearance in gingival biofilms.

At this time, however, we have reported a simpler approach. Dentifrices containing zinc are well established to reduce gingival inflammation and bleeding better than control dentifrices without zinc [169], but how zinc works has not been understood. Recently, we reported that zinc ions stop LdcE production at the *E. corrodens* cell surface, perhaps by attaching to a unique cysteine residue in its N-terminal region [170]. In addition, dietary zinc supplements promote systemic health by reducing systemic inflammation, indicating that zinc does not inhibit LdcI production in the gut [171]. The use of a new, well tolerated, zinc-containing dentifrice [172] could improve periodontal health without compromising intestinal health. Interestingly, stannous fluoride has also been reported to promote gingival health by inhibiting microbial SCFA production in the oral cavity [173]. Unfortunately, effects in the intestine are not known, despite the use of stannous fluoride in toothpaste for 20 years. Although only small amounts are swallowed, many years of using this toothpaste twice daily in the oral cavity might increase susceptibility to intestinal inflammation by reducing butyrate production in the gut (Section 1.3).

### 4.2. Interactions with Other Chronic Inflammatory Diseases

Histone acetylation by HATs and their deacetylation by HDACs, such as the protein sirtuin 1, controls DNA methylation, a common epigenetic signal that inhibits gene transcription (Section 3.3). Normally, DNA methylation and demethylation curb DNA methyltransferase expression and activity so as to maintain a homeostatic balance that controls proinflammatory gene transcription and oxidative stress [174]. Interrupting this crosstalk disregulates homeostasis and potentially explains the association of periodontal disease with many other systemic infections that induce or exacerbate other chronic inflammatory diseases such as diabetes [175], atherosclerosis [176], rheumatoid arthritis [177], some cancers [178], adverse pregnancy outcomes [179], and Alzheimer’s disease [180].

Sirtuin 1 is a histone deacetylase that regulates a variety of cellular functions, such as genome integrity and cellular metabolism associated with longevity. It reduces the transcriptional activity of NF-κB, a mediator of many inflammatory processes including periodontal inflammation and alveolar bone loss. As already noted in Section 2.9, HDACi activation upregulates genes such as HMGB1 that promote cell death and necrosis in vitro [138]. Because high concentrations of butyrate in the oral cavity induce HDACi, sirtuin 1 control of proinflammatory gene transcription and oxidative stress should be reduced (dysregulated). Sirtuin production and serum levels should therefore decrease in periodontitis but increase after therapy, as recently reported [181]. More importantly, a change or deterioration in any chronic inflammatory condition will dysregulate sirtuin 1 homeostasis [182]. For example, a failure to treat periodontitis decreases serum sirtuin levels and increases the severity of type 2 diabetes [175], whereas a failure to treat diabetes likewise increases systemic inflammation, decreases serum sirtuin levels, and promotes a greater likelihood of periodontal tooth loss [12].

Although there is no evidence that periodontal inflammation increases cancer in the oral cavity, IBD increases the risk of colorectal cancer (CRC). As noted in Section 3.2, peristalsis varies butyrate concentrations so as to provide the energy for a properly intact intestinal epithelium that keeps inflammation minimal. Butyrate is a product of fiber fermentation and mediates the protective effect of dietary fiber against CRC. Indeed, adding butyrate to the diet or anally enhances this anti-neoplastic activity by activating HDACi and inducing HMBG1 [183]. More recently, in a different approach, investigators used resveratrol to activate the sirtuin 1 deacetylase. The investigators found that this activation of sirtuin 1 inactivates HDACs (Section 3.3) and enhances the butyrate-mediated HDACi/HMBG1 necrosis of colorectal cancer in mice [184].

### 4.3. IBD

DAT cells have no function beyond protection, whereas the intestinal epithelium has the additional function of nutrient and fluid transfer. The uptake of butyrate from the intestinal lumen and its catabolism by mitochondria provides the extra energy required to transfer nutrients from the small intestine lumen, or water and bile salts from the colon lumen, to capillaries (Section 1.3). The provision of butyrate reduces the need for food by maximizing the amount of nutrients for systemic use. However, the price is a need for rapid turnover because cells exposed to amounts of butyrate greater than 0.5 mM in the lumen or villi must be replaced before they become necrotic. As noted in the legend to Figure 1b, agents that irritate intestinal epithelial cell function let oxygen into the lumen, causing a bloom of *Proteobacteria* that replaces butyrate with lactate and uses nitrate as an electron acceptor. The result is an excessive production of reactive nitrogen species that continues to impair the microbial epithelial barrier unless the dysbiotic microbiome is removed.

A current therapy for IBD is intravenous infusions of an anti-TNF drug such as ustekinumab to retard the host’s intestinal innate immune response (Section 3.1). This therapy is effective for some patients, but 30% are intractable and the anti-inflammatory effect decreases annually in another 20% of patients [149]. In view of the influence of butyrate on intestinal health, a relatively old suggestion being revised is to combine the anti-TNF therapy with butyrate intake, either directly or by ingesting bacteria important for providing butyrate [149]. Although butyrate or commensal bacteria that produce butyrate may be applied in enemas, most adults prefer the oral route. A study in humans assessed modifications of the intestinal microbial ecosystem after healthy adults had orally consumed *Bifidobacterium* *bifidum* [185]. This strictly anaerobic bacterium is found in whole grains and yogurt and has long been touted as a dietary supplement to benefit gastrointestinal health by reducing lactate to butyrate instead of excreting lactate. A double-blind, placebo-controlled crossover trial reported that a single daily administration of a *Bifidobacterium bifidum* strain enhanced the health of the intestinal microbial ecosystem in adults, as indicated by fecal microbiome analysis and the increased SCFA content in wet feces [186]. In conclusion, the oft-suggested change in our current diet to a dietary fiber-rich diet may facilitate the increased colonization of the intestine by butyrate-producing intestinal bacteria and reduce the incidence of both IBD and CRC.

## 5. General Conclusions

Figure 1 is a graphical abstract that summarized the key points of this review in Section 1. We have shown that the loss of an epithelial barrier plays a key pathogenic role in both periodontitis and IBD, and that oral hygiene in the oral cavity plays the same role as peristalsis in the intestines. In both diseases, microbially derived lysine decarboxylase, cadaverine production, and butyrate are critical effectors but act in opposite ways. In the oral cavity, the LdcE-mediated removal of lysine by conversion to cadaverine impairs the dentogingival microbial barrier, and the introduction of cadaverine stabilizes the later development of a dysbiotic microbiome. The latter microbiome hydrolyzes proteins in the stimulated GCF, and metabolizes them to butyrate, which destroys the periodontium. In the intestines, the LdcI production of cadaverine selects for probiotic bacteria that survive the acidic stomach and then ensures the development of a butyrate-producing microbiome that keeps the intestinal epithelium intact and free of inflammation. A combination of mucous secretion and peristalsis limits the intestinal butyrate to small concentrations in crypts where it activates growth, and large amounts in intestinal villi or the colon lumen where older cells are removed (anoikis). Zinc ions inhibit the LdcE activity responsible for oral but not intestinal microbial butyrate production. Although stannous fluoride inhibits oral butyrate production, it is uncertain whether it also inhibits intestinal butyrate production.

## Figures and Tables

**Table 1:** Changes in biofilm lysine and cadaverine before and after a week of EG.

Assay	Unit	Before EG ^a^	After EG ^a^
Biofilm	mg (s.d.) ^b^	-	-
^c^ Lys	µmol/g (s.d.)	0.17 (0.05)	0.07 (0.07) ^d^
^c^ Cad	µmol/g (s.d.)	0.21 (0.13)	0.34 (0.25) ^d^
Lys + Cad	µmol/g (s.d.)	0.38 (0.14)	0.41 (0.29)
^c^ CF	ratio (s.d.)	0.51 (0.14)	0.82 (0.11) ^d^

^a^ EG, experimental gingivitis for 1 week. ^b^ s.d., standard deviation. ^c^ Lys, Lysine; Cad, Cadaverine; CF, cadaverine fraction = Cad/(Lys + Cad). ^d^ Significant decrease in Lys, and increase in both Cad and CF after EG (respective Wilcoxon matched-pairs signed-rank tests *p* < 0.05). This Table is original, and made for this review using data from Lohinai et al. [73].

**Table 2:** Changes in biofilm lysine and cadaverine content in good and poor responders to periodontal therapy, comparison with healthy volunteers and untreated patients.

Group	Unit	^a^ GR	^a^ HV	^a^ PR	^a^ Untreated
Number	-	9	6	7	33
Biofilm	mg (s.d.) ^b^	16.0 (9.4)	15.0 (0.38)	20.2 (20.7)	13.4 (10.2)
Lys	µmol/g (s.d.)	0.19 (0.10)	0.20 (0.09)	0.07 (0.03) ^c^	0.25 (0.19)
Cad	µmol/g (s.d.)	0.17 (0.05)	0.15 (0.06)	0.08 (0.09) ^d^	0.11 (0.11)
Lys + Cad	µmol/g (s.d.)	0.36 (0.10)	0.35 (0.13)	0.15 (0.09) ^e^	0.36 (0.25)
CF	ratio (s.d.)	0.50 (0.15)	0.44 (0.44)	0.43 (0.23)	0.32 (0.19) ^f^

^a^ GR, good responders to therapy; HV, healthy volunteers; PR, poor responders to therapy, and untreated patients seeking treatment. ^b^ Mean amounts of biofilm sampled were similar for all 4 groups (Kruskal–Wallis test *p* > 0.05). ^c^ Lys in PR was less than in the other three groups (Kruskal–Wallis *p* < 0.01, two-tailed multiple pairwise comparisons < 0.02). ^d^ Cad did not differ between the groups (Kruskal–Wallis test *p* > 0.05). ^e^ Lys + Cad in PRs was less than in GRs and HVs, omitting the untreated group (Kruskal–Wallis: *p* < 0.02, two-tailed multiple pairwise comparisons: *p* < 0.04). ^f^ CF measurements were reduced only in the untreated group (Kruskal-Wallis: *p* < 0.02, two-tailed multiple pairwise comparisons: *p* < 0.05). Reprinted with permission from [3]. Copyright 2017 American Academy of Periodontology. Units for Lys and Cad are corrected here to read µmoL/g biofilm, same units as Table 1.

## Data Availability

Not appropriate for a review as the data, including our own, are already in the literature and have been referenced.
